# Patients’ sexual harassment of nurses and nursing students: A cross-sectional study

**DOI:** 10.1016/j.ijnsa.2023.100121

**Published:** 2023-03-07

**Authors:** Milena Marta Bruschini, Hannele Hediger, Ada-Katrin Busch

**Affiliations:** Institute of Nursing, Zurich University of Applied Sciences, Katharina-Sulzer-Platz 9, Winterthur, 8400, Switzerland

**Keywords:** Cross-sectional studies, Nurses, Patients, Prevalence, Sexual harassment, Switzerland, Workplace

## Abstract

****Background**:**

Workplace sexual harassment towards nurses is a global phenomenon: approximately one quarter of all nurses are affected by sexual harassment. The extent and type of sexual harassment vary greatly depending on the country, culture, level of education, and care setting. Notably, patients are amongst the main perpetrators. Importantly, sexual harassment has serious consequences on nurses’ health and work performance.

****Objective**:**

This study examined the prevalence of patients’ workplace sexual harassment towards nurses and nursing students at a University of Applied Sciences in Switzerland.

****Design**:**

A quantitative cross-sectional descriptive correlational design was used.

****Setting**:**

The survey was conducted at the university's Institute of Nursing under the Department of Health.

****Participants**:**

Nurses and nursing students who were studying or attending a continuing education programme and had worked as a nurse with direct contact with patients in the last 12 months could qualify as participants. A final sample of 251 participants was used for the analysis.

****Methods**:**

Data were collected using the ‘Sexually Harassing Behaviour Questionnaire from an extraorganizational perspective’. Preliminary analysis involved computing the percentage and absolute frequencies, mean scores, standard deviations, and ranges. The hypotheses were tested using non-parametric tests, such as the Wilcoxon test for two independent samples, Spearman correlation test, and Kruskal-Wallis H test. Results were considered statistically significant at alpha < 0.05.

****Results**:**

Most participating nurses were women (88.5%) and worked in adult acute care (54.2%). The mean age was 25.5 (*SD* = 7.5) years. On average, they had worked in the nursing profession for 7 years. 17.1% of the participants had received training on sexual harassment. Overall, 95.6% of the participants reported experiencing sexual harassment of any type at least once in the last 12 months. The most common type of harassment was verbal sexual harassment. Notably, sexual harassment was statistically significantly more frequent the younger the nurses were [*r_s_* = -0.13, *p* = 0.046]. Furthermore, it was statistically significantly more prevalent in adult acute care than in paediatrics [*H* (10) = 18.4; *p* = 0.048; Cohen's *d* = 0.4].

****Conclusions**:**

Patients’ sexual harassment of participant nurses and nursing students is highly common. The high prevalence of sexual harassment and low number of nurses who have received training on sexual harassment demonstrate the need for initiatives to address this phenomenon in the basic education of nurses. Furthermore, evidence-based interventions against sexual harassment in the nursing profession are needed.

## 1 Background

Globally, nurses are affected by sexual harassment in the workplace (Lu et al., 2020). Sexual harassment can be defined as any behaviour with a sexual reference, or based on gender that is unwanted from one side and violates the dignity of the person in question (Eidgenössisches Departement des Inneren [EDI] & Eidgenössisches Büro für die Gleichstellung von Frau und Mann (Eidgenössisches Departement des Inneren [EDI] and Eidgenössisches Büro für die Gleichstellung von Frau und Mann [EBG], 2008). This phenomenon can be divided into three types: nonverbal, verbal, and physical sexual harassment (Vincent-Höper et al., 2020).

Compared with other professions, nurses are more likely to experience sexual harassment in the workplace. This is because nurses’ daily work involves substantial physical contact; further, they often interact with people who are under great stress or in a state of intoxication (Celik and Celik, 2007; Cheung et al., 2017; Nielsen et al., 2017). According to Spector et al. (2014), approximately 25% of nurses worldwide are affected by workplace sexual harassment. Almost 70% of nurses affected by sexual harassment report negative psychological and physical consequences, including anxiety, humiliation, frustration, gastrointestinal discomfort, headaches, insomnia, and exhaustion. Furthermore, multiple studies have confirmed the negative effects of workplace sexual harassment on nurses’ work performance and quality of care (Celik and Celik, 2007; Cogin and Fish, 2009; Maghraby et al., 2020; Nielsen et al., 2017). Nevertheless, sexual harassment in the workplace often goes unreported. Along with saying no and avoiding the patient, doing nothing is one of the most common strategies that nurses use against sexual harassment (Celik and Celik, 2007; Cogin and Fish, 2009; Nielsen et al., 2017). Nurses often feel that reporting will not have any consequences or are too ashamed to talk about it (Kim et al., 2018; Nelson, 2018).

In addition, there is a lack of guidelines, evidence-based interventions, and preventive measures against workplace sexual harassment (Fujimoto et al., 2019; Koller and Spörhase, 2018; Nielsen et al., 2017). Furthermore, there are few education programmes for nurses on workplace sexual harassment (Adler et al., 2021; Kim et al., 2018; Zeng et al., 2020). According to Kim et al. (2018) and Nielsen et al. (2017), education on sexual harassment can help nurses develop individual coping strategies and counter the feelings of being overwhelmed or insecure.

Social media initiatives, such as #MeToo, #TimesUp, and #EndNurseAbuse, which were initiated in 2017 and 2018, brought attention to the issue of sexual harassment at the workplace. These initiatives have helped to raise awareness about the issue of sexual harassment so that nurses are better able to recognise and report it. Early recognition and reporting of sexual harassment are important to prevent abuse from leading to even more serious crimes such as physical injuries or rape and can thereby improve the safety of nurses (McClendon and Farbman, 2018; Nelson, 2018).

The prevalence of sexual harassment and its perpetrators differ by country, region, culture, and care setting (Lu et al., 2020; Spector et al., 2014). In research from the Global North, such as Switzerland, sexual harassment most often originates from patients (Cogin and Fish, 2009; Magnavita and Heponiemi, 2011; Nielsen et al., 2017; Park et al., 2015). In contrast, in the Global South, other groups of perpetrators can be more prevalent, such as co-workers, patients' relatives, or physicians (Celik and Celik, 2007; Margavi et al., 2020; Tsukamoto et al., 2019). Here, the terms ‘Global North’ and ‘Global South’ describe more developed, richer countries and less developed, poorer countries, respectively. They replace the outdated terms ‘first-world countries’ and ‘third-world countries’ (Dados and Connell, 2012).

In addition, the frequency of sexual harassment differs depending on nurses’ educational level. For example, in some studies, the prevalence of sexual harassment differed between nurses with a bachelor's degree, and those with a vocational degree or assistant nurses (Celik and Celik, 2007; Hibino et al., 2009), and between nursing students and those who had completed nursing education (Cogin and Fish, 2009; Magnavita and Heponiemi, 2011).

Switzerland has a diverse and unique educational system for nursing professionals. There are many different training courses and jobs for nurses in the Swiss healthcare system. These training programmes are held at various educational levels and last between a few months (for short nursing courses) to two to four years (for a basic nursing training). In Swiss nursing practice, nurses play various roles and positions, each with a different set of competencies. These roles range from nursing interns to advanced practice nurses (APN) (Staatssekretariat für Bildung, Forschung und Innovation [SBFI] and Eidgenössisches Departement für Wirtschaft, Bildung und Forschung [WBF], 2016).

An extensive literature search revealed no isolated data on the prevalence of sexual harassment in Switzerland amongst nurses and nursing students in various care settings. Owing to the variance in study results worldwide and the great influence of culture, setting, and education on the occurrence of workplace sexual harassment towards nurses, there is a need to measure the prevalence and types of the same in Switzerland.

### 1.1 Aim

The primary aim of this study was to determine the prevalence of patients’ workplace sexual harassment towards nurses and nursing students at a University of Applied Sciences in Switzerland. The study sought to identify the risk factors associated with sexual harassment. The results of the study can be used to develop targeted evidence-based interventions.

### 1.2 Research question and hypotheses

To achieve our aim, we proposed the following research question: How often and which types of workplace sexual harassment were experienced by nurses and nursing students from patients at a University of Applied Sciences in Switzerland?

Furthermore, four hypotheses were formulated:

HA: The prevalence of sexual harassment differs amongst nurses who have and have not completed education.
HA: The prevalence of sexual harassment and the age of the participants are correlated.
HA: The prevalence of sexual harassment differs between different care settings.
HA: The prevalence of sexual harassment differs by the educational level of participants.

## 2 Methods

This study used a cross-sectional descriptive correlational design. Participants were recruited through convenience sampling. Finally, primary data were collected from participants via a questionnaire survey.

### 2.1 Participants and setting

Data collection was conducted at the Institute of Nursing, Department of Health, at one University of Applied Sciences in the German speaking part of Switzerland. The university offers full-time and part-time bachelor's and master's degree programmes for nursing professionals. In addition, it provides continuing education courses and programmes on various nursing-specific topics for registered nurses. In the autumn semester of 2021, 682 individuals were studying at the Institute of Nursing, who formed the population of this study. This study site and population are particularly suitable because the age, educational background, and place of work of nurses and nursing students are widespread. Further, the heterogeneity helps in mapping the phenomenon of sexual harassment towards nurses under different conditions and thus detect differences in prevalence of harassment.

Many nurses who study or attend continuing education course at a University of Applied Sciences have already completed basic nursing training, and are working part-time in nursing. Thus, not only nurses who are studying for a bachelor's or master's degree were included in the study, but also those who work as qualified nurses and are attending a continuing education or diploma course.

Care settings and types of nursing jobs were not limited. In Switzerland, paediatric departments treat children and adolescents up to puberty, ages 0–18 (Kind + Spital, 2016; Pädiatrische Pflege Schweiz, 2020). As the occurrence of sexual harassment amongst adolescents cannot be precluded, we did not exclude paediatric departments. Furthermore, participants were included regardless of their age, sex, and professional experience.

The precondition for participation was employment in nursing, with direct patient contact in the last 12 months. In addition, the participants had to understand German to complete the questionnaire.

Due to the sensitive nature of the topic, participation was voluntary and convenience sampling was used for recruitment. Therefore, it is not possible to generalise the results to the study population, other Universities of Applied Sciences in the German speaking part of Switzerland or all Swiss nurses and nursing students.

### 2.2 Data collection

The data were collected over 38 days in October and November 2021. The survey was conducted online using the electronic data collection tool REDCap. REDCap is a secure web application that guarantees full anonymity when participating (Harris et al., 2009, 2019).

Potential study participants were recruited through an online advertisement on the University of Applied Sciences**’** student website. In addition, flyers were displayed on the university's campus. On some days, nurses and nursing students were approached personally before, during, or after lectures to encourage participation.

Data were collected using the ‘Sexually Harassing Behaviour Questionnaire from an extraorganizational perspective’ (SHBQ-X) developed by Vincent-Höper et al. (2020) and adapted by Adler et al. (2021). The SHBQ-X instrument was used in the original German language and therefore did not require translation. According to Vincent-Höper et al. (2020) and Adler et al. (2021), the content and construct validity, as well as the reliability of this instrument has been confirmed for healthcare settings and the perpetrator group of patients (Cronbach's alpha = 0.80–0.92).

The SHBQ-X instrument contains 14 items and measures experiences of sexual harassment by patients, clients, or residents that occurred in the last 12 months. Overall, 12 items are related to sexual harassment, consisting of 4 items each on nonverbal, verbal, and physical sexual harassment. These items are answered using a six-point Likert scale from zero to five, with zero representing ‘never’ and five representing ‘(nearly) every day’. Items 13 and 14 covered attempted rape and forced sexual acts, and were responded as ‘yes’ or ‘no’.

Further, participants’ demographic information was collected, including gender, age, last nursing job position and care setting, number of years of work experience in nursing, highest completed education, shift work, and whether they had received sexual harassment training. To ensure that no conclusions can be drawn about individual persons, no reference was made to the academic year, the degree programme or the place of work. No other personal data such as name, telephone number or email was collected either.

Participants were asked to answer the survey completely to enable a comparison of the prevalence of different types of sexual harassment. If an item was forgotten, participants were shown a reminder from the online data collection tool before submitting.

### 2.3 Statistical analysis

REDCap automatically created a dataset with all collected variables. Following Leonhart (2010), these data were then checked for potential errors and illogical values. Finally, data analysis was performed using R version 4.0.1 and RStudio version 1.3.1093.

For the three types of sexual harassment in the SHBQ-X instrument, three subscales were formed from the 4 items of each type, one for nonverbal, verbal, and physical sexual harassment, respectively. All 12 items together formed the overall scale. To create these scales, only data from participants who had answered all 12 items on sexual harassment could be analysed.

Percentage and absolute frequencies, mean scores, standard deviations, medians, and ranges were determined for the demographic data, depending on the scale level. Furthermore, the percentage and absolute frequencies were calculated for each of the 12 different types of sexual harassment.

To calculate the percentage and absolute frequencies of the overall scale and the three subscales of sexual harassment and to ensure that the results were comparable with other studies’ findings, all items were dichotomised. This implied that if one participant answered at least one item of the overall scale or subscales with at least ‘once in 12 months’, it was considered that this person had experienced sexual harassment in general or this type of sexual harassment in the past year.

In addition, the mean score, standard deviation, median, and range were calculated for the overall and subscale scores of sexual harassment. The mean score for each participant was calculated by adding the scores with possible values between zero and five of the individual items, all 12 for the overall scale and 4 for each of the subscales. Subsequently, the sum was divided by the number of items. The higher the overall average or the subscale score, the more often the participants were affected by various types of sexual harassment.

Next, whether the data were normally distributed was tested using the Shapiro-Wilk test. As the variables were not normally distributed, hypothesis testing was performed using non-parametric tests, such as the Wilcoxon test for two independent samples, Spearman correlation test, and Kruskal-Wallis H test. The overall average score of sexual harassment, with possible values from zero to five, was used as the dependant variable.

Results were considered statistically significant at alpha < 0.05. For statistically significant results, effect sizes were calculated according to Cohen (1988) using Psychometrica (Lenhard and Lenhard, 2016). Cohen (1988) has categorised the effects as small (*d* = 0.20), medium (*d* = 0.50), and large (*d* = 0.80).

Cronbach's alpha was used to demonstrate internal consistency for both the overall scale and subscales. A Cronbach's alpha ≥ 0.80 is considered excellent (Cronbach, 1960).

### 2.4 Ethics approval and consent to participate

Participation in the study was voluntary, and the participants had to provide informed consent. Anonymity was guaranteed during the data collection.

This study was approved by the responsible leaders of the Department of Nursing at the University of Applied Sciences. Furthermore, the planned project was submitted to the Cantonal Ethics Committee Zurich, and a confirmation of non-responsibility was given (BASEC—Nr. Req-2021–00886).

## 3 Results

Out of the possible 682 participants, a total of 281 nurses and nursing students from the chosen University of Applied Sciences participated in the survey; 30 respondents were excluded as they either did not provide consent (*n* = 1), had not worked in the care sector in the last 12 months (*n* = 5), or had answered the questionnaire on sexual harassment incompletely (*n* = 24). Finally, the responses of 251 participants were evaluated (response rate: 36.8%). The Cronbach's alphas for the overall scale and subscales of sexual harassment were between 0.80–0.90.

### 3.1 Sample description

The sociodemographic characteristics are summarised in Table 1. Most participating nurses were women (88.5%). All participants were between 18 and 58 years of age, with an average age of 25.5 years. In total, 91 (36.3%) participants reported that their most recent professional nursing role was as a nurse intern, nurse in training, or a nursing student. Thus, these 91 participants were counted as nurses who had not completed their nursing education at the time the study was conducted. The remaining 160 (63.7%) participants reported that they had completed nursing education and were counted as such. The mean age and work experience of participants who had not completed education was 21.5 (*SD* = 3.0 years) and 3.2 (*SD* = 2.6 years) years, respectively, compared with 27.8 (*SD* = 8.3 years) and 9.4 (*SD* = 7.2 years) years, respectively for participants who had a nursing degree. Work experience in years was counted including the collected experience in nursing during the nursing training and could be stated exactly to two decimal places. Most participants worked in an adult acute care setting (54.2%), followed by long- term care (9.6%). Further, 92.4% participants stated that they worked in shifts, 17.1% had received training on sexual harassment.

**Table 1:** Demographic information of participants.

Total *n* = 251		
Gender, *n* (%) Women Men Diverse	222 28 1	(88.5) (11.2) (0.4)
Age in years, *M ± SD; Mdn* (min.–max.)	25.5 ± 7.5; 24 (18–58)	
Position, *n* (%) Nursing internship Nurses in training Nursing student Nursing assistant Licenced practical nurse (LPN) Registered nurse (RN) RN with postgraduate diploma RN with specialist responsibility or RN with additional function Nurses with a vocational training function Nursing expert or advanced practice nurse (APN) Nurse with management function Other	16 23 52 13 34 57 9 27 5 8 6 1	(6.4) (9.2) (20.7) (5.2) (13.5) (22.7) (3.6) (10.8) (2.0) (3.2) (2.4) (0.4)
Setting, *n* (%) Adult acute care inpatient Emergency Intensive care Anaesthesia or surgery or recovery room Long-term care inpatient Psychiatry inpatient Home visit nursing Outpatient care Rehabilitation Paediatrics Other	136 5 5 6 24 18 14 11 9 16 7	(54.2) (2.0) (2.0) (2.4) (9.6) (7.2) (5.6) (4.4) (3.6) (6.4) (2.8)
Work experience in nursing in years, *M ± SD; Mdn* (min.–max.)	7.1 ± 6.6; 5.3 (0.1–40)	
Highest completed education, *n* (%) Vocational training High-school education Federal diploma of higher education Bachelor Federal diploma of higher education or bachelor with additional education Master Doctorate Other	7 125 26 68 20 5 - -	(2.8) (49.8) (10.4) (27.1) (8.0) (2.0) - -
Shift work, *n* (%) Yes No	232 19	(92.4) (7.6)
Received training on sexual harassment, *n* (%) Yes No	43 208	(17.1) (82.9)

Note. *n* = sample size; *M* = mean score; *SD* = standard deviation; *Mdn* = median; min.–max. = minimum and maximum.

### 3.2 Prevalence of sexual harassment in nursing

The prevalence of overall sexual harassment and of the three subscales in the last 12 months and Cronbach's alpha values are presented in Table 2. The frequencies and types of sexual harassment according to the 12 SHBQ-X items are shown in Table 3.

**Table 2:** Prevalence of the overall scale and subscales of sexual harassment.

			Total *n* = 251	
	***n* (%) experienced sexual harassment at least once in 12 months**	**Score**	***M* ± *SD, Mdn*** **(min.-max.)**	**Cronbach's α**
Overall scale of sexual harassment	240 (95.6)	0–5	1.0 ± 0.7, 0.9 (0–3.8)	0.9
Subscale of types of sexual harassment				
Nonverbal sexual harassment	193 (76.9)	0–5	0.7 ± 0.7, 0.5 (0–4.5)	0.8
Verbal sexual harassment	234 (93.2)	0–5	1.7 ± 1.1, 1.8 (0–5)	0.9
Physical sexual harassment	171 (68.1)	0–5	0.6 ± 0.7, 0.5 (0–3.3)	0.8

Note. *n* (%) = Number and percentage of participants who were sexually harassed at least once in the last 12 months; Score = 0 ‘never’ to 5 ‘(nearly) every day’; *M* = mean score; *SD* = standard deviation;

*Mdn* = median; min.–max. = minimum and maximum; Cronbach's α = Cronbach's alpha.

**Table 3:** Frequencies of the different types of sexual harassment assessed with the SHBQ-X questionnaire.

					Total *n* = 251	
Type of sexual harassment	**Never**	**Once in 12 months**	**Every few months**	**Every few weeks**	**Every few days**	**(Nearly) every day**
*Nonverbal sexual harassment*						
I have witnessed sexual acts	157 (62.5)	63 (25.1)	22 (8.8)	6 (2.4)	3 (1.2)	0 (0)
I have witnessed sexual gestures	74 (29.5)	81 (32.3)	58 (23.1)	31 (12.4)	6 (2.4)	1 (0.4)
Someone has unnecessarily exposed themselves in front of me	169 (67.3)	51 (20.3)	20 (8.0)	10 (4.0)	0 (0.0)	1 (0.4)
I have witnessed sexual harassment/violence amongst patients/clients/residents	189 (75.3)	37 (14.7)	17 (6.8)	4 (1.6)	3 (1.2)	1 (0.4)
*Verbal sexual harassment*						
I have been sexually complimented	29 (11.6)	44 (17.5)	82 (32.7)	61 (24.3)	29 (11.6)	6 (2.4)
I have been told suggestive/offensive stories or jokes	40 (15.9)	58 (23.1)	74 (29.5)	48 (19.1)	27 (10.8)	4 (1.6)
I have been exposed to verbal sexual innuendo	66 (26.3)	54 (21.5)	74 (29.5)	42 (16.7)	13 (5.2)	2 (0.8)
I have been asked intrusive or personal questions by a client (e.g. requests for body measurements, relationship status, or sexual preferences)	91 (36.3)	64 (25.5)	52 (20.7)	27 (10.8)	15 (6.0)	2 (0.8)
*Physical sexual harassment*						
I have been hugged in a way that made me feel uncomfortable	166 (66.1)	57 (22.7)	18 (7.2)	7 (2.8)	3 (1.2)	0 (0.0)
I have been petted or patted in a way that made me feel uncomfortable	122 (48.6)	72 (28.7)	42 (16.7)	9 (3.6)	6 (2.4)	0 (0.0)
I have been touched in a way that made me feel uncomfortable	120 (47.8)	88 (35.1)	27 (10.8)	8 (3.2)	7 (2.8)	1 (0.4)
I have been kissed in a way that made me feel uncomfortable	190 (75.7)	44 (17.5)	16 (6.4)	0 (0.0)	1 (0.4)	0 (0.0)

Note. *n* = sample size, (%).

Overall, 240 (95.6%) participants reported experiencing sexual harassment of at least one type at least once in the last 12 months. In addition, one person each reported attempted rape and being forced to perform sexual acts (0.4%). No experience of workplace sexual harassment by patients in the last 12 months was reported by 11 participants (4.4%).

The most common type was verbal sexual harassment; 93.2% of the participants reported experiencing it at least once in the last 12 months (*M* = 1.7, *SD* = 1.1). This was followed by nonverbal sexual harassment, reported by 76.9% of the participants (*M* = 0.7, *SD* = 0.7). Finally, physical sexual harassment was experienced by 68.1% of the participants (*M* = 0.6, *SD* = 0.7).

The participants were most likely to receive sexual compliments (88.4%, *M* = 2.1, *SD* = 1.2). The least frequent was being kissed (or attempted to be kissed) by patients in a way that made participants feel uncomfortable (24.3%, *M* = 0.3, *SD* = 0.6).

### 3.3 Comparison of frequencies of sexual harassment based on demographics

The Wilcoxon test showed no statistically significant difference in the prevalence of sexual harassment amongst nurses who had completed a nursing degree, and those still in training or studying (*W* = 7104; *p* = 0.751). Therefore, the alternative hypothesis was not accepted.

**Fig. 1:**
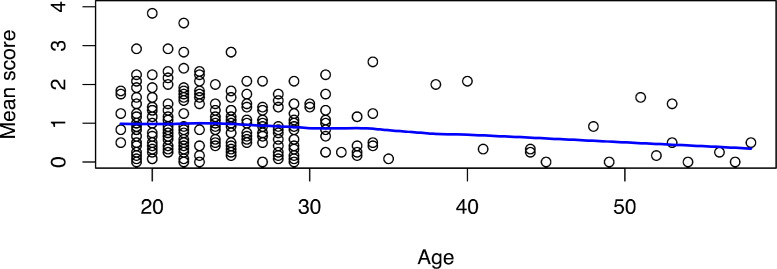
Scatter plot relationship between age and prevalence of sexual harassment, *n* = 251 Note. Age in years, mean score of sexual harassment scale (0 = ‘never’ and 5 = ‘(nearly) every day’).

The Spearman's correlation test revealed a statistically significant relationship between participants’ age and the prevalence of sexual harassment (*r_s_* = −0.13; *p* = 0.046). The correlation was weak and negative. The younger the participants, the more frequent was the sexual harassment (Fig. 1).

The Kruskal-Wallis H test revealed a statistically significant difference in the prevalence of sexual harassment in different care settings (*H* (10) = 18.4; *p* = 0.048; Cohen's *d* = 0.4).

The Bonferroni post-hoc-analysis showed that there were significant differences between paediatrics and adult acute care (*p* = 0.027; see Table 4), but no significant differences between other care settings.

**Table 4:** Post-hoc-analysis: Significant differences in the prevalence of sexual harassment and care settings.

	Paediatrics (*n* = 16)	Adult acute care (*n* = 136)	p-value
Prevalence of sexual harassment *M* ± *SD* (min.–max.)	0.4 ± 0.5 (0–1.7)	1.0 ± 0.7 (0–3.8)	0.027

Note. *n* = sample size; *M* = mean score; *SD* = standard deviation; min.–max. = minimum (0) and

maximum (5).

In paediatrics, 68.8% of the respondents experienced at least one type of sexual harassment at least once in the last 12 months (*M* = 0.4, *SD* = 0.5), compared to 97.8% in adult acute care settings (*M* = 1.0, *SD* = 0.7). There was a small effect based on Cohen's *d* with 0.4.

The Kruskal-Wallis H test showed no statistically significant difference in the prevalence of sexual harassment across different educational level of nurses (*H* (5) = 4.0; *p* = 0.545). Therefore, the alternative hypothesis was not accepted.

## 4 Discussion

This study explored the prevalence of nurses’ and nursing students’ workplace sexual harassment by patients at a University of Applied Sciences in the German speaking part of Switzerland, as well as its risk factors. Data from 251 respondents were collected via an online questionnaire survey—consisting of Vincent-Höper et al. (2020)'s SHBQ-X questionnaire and adapted from Adler et al. (2021), as well as demographic elements—conducted at the university's Institute of Nursing.

The results showed that nurses and nursing students regularly experience workplace sexual harassment: almost all participants (95.6%) had been sexually harassed by the patients at least once in the last 12 months. Amongst the three types of sexual harassment, verbal sexual harassment was the most common, followed by nonverbal and then physical sexual harassment.

Most participants (88.5%) were women, reflecting the normal gender distribution of the nursing profession in Switzerland (Bundesamt für Statistik [BFS], 2020). The mean age of the participants was 25.5, which is much lower than the Swiss average age of nurses at 45.5 years (BFS, 2020). Finally, the age range in this study was 18–58 years. Note that in Switzerland, the retirement age is 64 years for women and 65 years for men (Zentrale Ausgleichsstelle [ZAS], 2019). Thus, this study did not cover the last 6 to 7 years of employment. At Swiss universities of applied sciences, the average age at entry is 23.1 years and at graduation, 26.7 years for a bachelor's degree, and 29.5 for a master's degree (BFS, 2018). Thus, the average age of participants of this study corresponds with that for students at other Swiss universities of applied sciences.

At 95.6% (*n* = 251), the overall prevalence of sexual harassment in the last 12 months is high, compared to other studies. For example, a review by Spector et al. (2014) and a meta-analysis by Lu et al. (2020) reported a prevalence of sexual harassment of 17.9% (*n* = 65,424 nurses) and 12.6% (*n* = 52,345 nurses) in the last 12 months, respectively. Note that this study evaluated the overall prevalence of sexual harassment as experiencing at least one type of sexual harassment at least once in the last 12 months. Furthermore, it assessed three types of sexual harassment (nonverbal, verbal, and physical). Meanwhile, Lu et al. (2020) and Spector et al. (2014) based their findings on other studies, with each sampled study using different measurement methods and definitions of sexual harassment. Lu et al. (2020) and Nielsen et al. (2017) highlight that there has not yet been a universally accepted instrument to assess and recognise sexual harassment. Therefore, the comparability of the prevalence of sexual harassment with other studies is limited. However, as Adler et al. (2021) used the same measurement instrument and similar data analysis methods as in this study, we can compare the results regarding the different types of sexual harassment. In Adler et al. (2021), the prevalence of sexual harassment compared to that in this study was slightly lower, that is 67.1% versus 93.2% for verbal, 62.5% versus 76.9% for nonverbal, and 48.9% versus 68.1% for physical sexual harassment, respectively. These lower values may be because apart from nurses (60%), the aforementioned study also included other healthcare staff. Nurses are more likely to experience sexual harassment from patients than other health workers because they spend more time with patients and more often perform tasks with close physical contact, which is a risk factor for harassing behaviour (Celik and Celik, 2007; Cheung et al., 2017; Nielsen et al., 2017). Previous single studies that also showed much higher prevalence of sexual harassment in comparison to the review by Spector et al. (2014) and the meta-analysis by Lu et al. (2020) were the ones from Cogin and Fish (2009) with 56% (*n* = 538), Hibino et al. (2009) with 56% (*n* = 464) and Maghraby et al. (2020) with 58% (*n* = 296). These three studies, like the one presented, used a measurement tool that not only determined the prevalence of sexual harassment using a definition with a yes and no question, but also provided examples of different types of sexual harassment. In addition, they also counted sexual harassment as prevalent if at least one type of sexual harassment was experienced and included no other healthcare personnel than nurses.

Further, this study showed that the younger the nurses were, the more frequently they experienced sexual harassment. This finding is also supported by Hibino et al. (2009), Lu et al. (2020), and Tsukamoto et al. (2019). One reason for the higher prevalence of sexual harassment in this study could be the relatively low average age of the participants compared to other studies (Lu et al., 2020). Hibino et al. (2009) stated that younger nurses are more aware of sexual harassment and thus are more likely to recognise and report it. In addition, Nielsen et al. (2017) described that with increasing age and longer work experience, some nurses no longer consider sexual harassment as threatening and even perceive it as less frequent.

In general, the prevalence of sexual harassment in studies was higher when they focused only on the phenomenon of sexual harassment towards nurses and did not assess other types of violence (Adler et al., 2021; Cogin and Fish, 2009; Hibino et al., 2009; Maghraby et al., 2020). This was also the case in the present study, and may suggest the high prevalence of sexual harassment.

Another reason for the high prevalence of sexual harassment could be the questionnaire employed. Before the SHBQ-X questionnaire was developed, validated measures that assess different types of sexual harassment experienced by employees working with patients or clients were lacking. The SHBQ-X questionnaire measured the occurrence of sexual harassment very sensitively. It captured many different types of sexual harassment and gave examples of harassing behaviour.

The #MeToo, #TimesUp, and #EndNurseAbuse initiatives on social media may also have contributed to the sensitisation of nurses to sexual harassment at the workplace and influenced the increased numbers in this study (McClendon and Farbman, 2018; Nelson, 2018).

Next, verbal sexual harassment was the most common type of harassment (93.2%). This is consistent with Celik and Celik's (2007) observation at 83.5%, Maghraby et al. (2020) at 53.5%, and Adler et al. (2021) at 67.1%. A Swiss study examining the forms of violence in Swiss psychiatric wards found that verbal sexual violence was more common (39%) than physical sexual violence (14%; Schlup et al., 2021). In Margavi et al. (2020), the majority of participants reported not reporting verbal abuse, whereas physical harassment was mostly reported to superiors. One reason for this difference could be that physical (sexual) violence is perceived as more threatening and more likely to lead to serious injuries (Gabrovec, 2017; Nielsen et al., 2017; Schlup et al., 2021). This demonstrates that, in preventing sexual harassment, the focus must not only be on the quantity but also on the quality of the incidents.

In this study, only 17.1% of the participants reported receiving training on sexual harassment. Other studies have also reported a lack of education or sensibilisation on this topic. For example, Adler et al. (2021) reported 29.3% of the respondents having attending training on sexual harassment. In Maghraby et al. (2020), no participant reported attending education sessions on workplace sexual harassment. In Kim et al. (2018), the participants, all of whom were nursing students, stated that because of the lack of education on this topic, they felt unprepared and did not know how to react to sexual harassment. This shows that regular training programmes on sexual harassment in care institutions are crucial (Adler et al., 2021; Lu et al., 2020). This phenomenon should be addressed in the basic training of nurses. Nurses need to understand that sexual harassment is not a part of the professional profile of nursing, even before they learn specific forms of coping and intervention (Celik and Celik, 2007; Nielsen et al., 2017; Zeng et al., 2020). Notably, Koller and Spörhase (2018) and Nielsen et al. (2017) clearly pointed out that talking to a person with trust and breaking the taboo regarding sexual harassment is an important coping strategy, and it helps nurses to develop personal strategies. In addition, actively addressing sexual harassment helps develop a zero-tolerance policy against abuse at the workplace (McClendon and Farbman, 2018; Temkin et al., 2022; Zeng et al., 2020).

In this study, more than half of the participants worked in adult acute care, which is slightly above the average of 45% of all Swiss nurses (BFS, 2020). According to Spector et al. (2014), the frequency of sexual harassment varies depending on the care setting. This study found similar results: the prevalence of sexual harassment differed significantly in adult acute care versus paediatrics. In Switzerland, a nurse in paediatrics caters to children who are in transition to adulthood (Pädiatrische Pflege Schweiz, 2020). Sexual desires emerge during early puberty. For example, 11–12-year-old boys and girls report that they frequently think about sex (Fortenberry, 2013). However, note that most of the hospitalised children in Switzerland are under the age of five (BFS, 2014). This may explain why the occurrence of sexual harassment in this setting is relatively low (68.8%) compared to other settings, such as adult acute care (97.8%).

Furthermore, this study found no correlation between nurses’ education level and the frequency of harassment. In contrast, several studies have shown that nurses with a higher education level are more likely to experience sexual harassment (Celik and Celik, 2007; Hibino et al., 2009). Finally, no statistically significant differences were observed in the prevalence of sexual harassment amongst nurses with a nursing degree and those still in training. This result differs from the findings of other studies. For example, Cogin and Fish (2009) reported that the prevalence of sexual harassment was noticeably higher amongst nursing students than amongst nurses who had completed their education. In contrast, Magnavita and Heponiemi (2011) found that nurses who had completed education experienced more sexual harassment than nursing students. This is despite the fact that in this study, as in the studies by Cogin and Fish (2009) and Magnavita and Heponiemi (2011), the age and work experience of nursing students were much lower than those with a nursing degree.

In summary, this study showed that sexual harassment is highly prevalent amongst nurses and nursing students at one of the Universities of Applied Sciences in the German speaking part of Switzerland. All three types of sexual harassment (nonverbal, verbal, and physical) were experienced within the past year. Further, this study revealed that some factors, such as age and care setting, can increase the risk of sexual harassment.

### 4.1 Strengths and limitations

This study has some strengths. First, the study used the SHBQ-X to measure the prevalence of sexual harassment. The validity of this questionnaire has been tested on a sample similar to that used in the present study and therefore is transferable (Adler et al., 2021; Vincent-Höper et al., 2020). Second, this study had a relatively high response rate of 37%.

Nevertheless, this study also has some limitations. As convenience sampling was used, there is a possibility of selection bias, and the results cannot be generalised to the population. Consequently, it is possible that mainly those nurses familiar with the problem of sexual harassment participated in this study and thus influenced its prevalence. In addition, participants were recruited via displayed study flyers on campus and advertisements on the student website. As the campus and the student website are open to the public, nurses who are not yet or no longer studying, or attending a continuing education programme at this University of Applied Sciences may have also participated in the study. Furthermore, we cannot exclude the possibility that some participants may have participated more than once in the survey. Another limitation is that this study only depicted the occurrence of sexual harassment in one university of applied sciences in the German-speaking part of Switzerland which is only one Swiss cultural area. This sample, with its different nursing professions and average age, did not represent the total population of nurses in the Swiss healthcare system. Therefore, the transferability of the results to other areas of Switzerland or the Swiss healthcare system is only partially possible.

## 5 Conclusions

Nurses and nursing students at the chosen University of Applied Sciences in Switzerland frequently experience workplace sexual harassment by patients. Worryingly, verbal sexual harassment affects some nurses on a weekly to (nearly) daily basis. Despite the high prevalence of sexual harassment, less than 20% of the participants had received sexual harassment training. This underscores the importance of addressing workplace sexual harassment in nurses’ basic training. Through education, nurses and nursing students can be better prepared and supported to deal with sexual harassment. Training should include how sexual harassment manifests in everyday working life and what factors contribute to it. Possible strategies on how to handle sexually harassing behaviour, why it is important to report it, and where to get help should also be addressed. It is paramount that the taboo surrounding nurse abuse is broken, and that it is universally recognised that sexual harassment is not part of the professional profile of nursing and must not be tolerated. The government and healthcare managers need to take measures to increase zero tolerance of violence and harassment in the workplace. Therefore, every healthcare facility must establish clear policies for dealing with sexual harassment and managers must ensure that their employees are aware of these policies. Patients’ sexual harassment in nursing still has hardly any consequences for the perpetrators, as it is rarely reported. It is advisable that zero tolerance of harassment is clearly communicated and that violations of the rules are punished. Not only nurses need to be educated about zero tolerance of violence and harassment, but also patients need to know about it. This is the basis for creating a respectful working environment free from harassment of any kind.

In this study, as most participants worked in adult acute care, other large nursing settings, such as long-term care or home visit nursing, were underrepresented. Further research in these areas is required to understand the occurrence of this phenomenon in these settings.

Finally, this study argues to ensure comparability of results and trends in the prevalence of sexual harassment, consistent measurement methods and definitions for sexual harassment should be used.

In summary, evidence from this study demonstrate the serious need for evidence-based interventions against sexual harassment in the nursing profession.

What is already known

• Globally, nurses are affected by sexual harassment by patients in their workplace.
• Sexual harassment negatively affects nurses’ health and work performance.
• The prevalence of sexual harassment differs by country, region, culture, level of education, and care setting.

What this paper adds

• This Swiss study shows that almost all participant nurses have experienced patients’ sexual harassment at least once in the last 12 months.
• Sexual compliments, offensives jokes or stories, and sexual innuendo from patients are encountered by 30% of participating nurses monthly in their workplace.
• Education is lacking regarding sexual harassment at the workplace for nurses and nursing students in Switzerland, highlighting an urgent need for action.

## Funding sources

This research did not receive any specific grant from funding agencies in the public, commercial or not-for-profit sectors.

## Declaration of Competing Interest

The authors declare that there are no conflicts of interest.

## References

[bib0001] Adler M., Vincent-Höper S., Vaupel C., Gregersen S., Schablon A., Nienhaus A. (2021). Sexual harassment by patients, clients, and residents: investigating its prevalence, frequency and associations with impaired well-being among social and healthcare workers in Germany. Int. J. Environ. Res. Public Health.

[bib0002] Bundesamt für Statistik (2014). Kinder im Spital. BFS Aktuell.

[bib0004] Bundesamt für Statistik (2020). Pflegepersonal 2018. BFS Aktuell.

[bib0005] Celik Y., Celik S.S. (2007). Sexual harassment against nurses in Turkey. J. Nursing Scholarship.

[bib0006] Cheung T., Lee P.H., Yip P.S.F. (2017). Workplace violence toward physicians and nurses: prevalence and correlates in Macau. Int. J. Environ. Res. Public Health.

[bib0007] Cogin J., Fish A. (2009). Sexual harassment—A touchy subject for nurses. J. Health Organ. Manag..

[bib0008] Cohen J. (1988).

[bib0009] Cronbach L.J. (1960).

[bib0010] Dados N., Connell R. (2012). The Global South. Contexts.

[bib0011] Eidgenössisches Departement des Inneren, & Eidgenössisches Büro für die Gleichstellung von Frau und Mann (2008). Sexuelle Belästigung am Arbeitsplatz. Schweizerische Eidgenossenschaft.

[bib0012] Fortenberry J.D. (2013). Puberty and adolescent sexuality. Horm. Behav..

[bib0013] Fujimoto H., Greiner C., Hirota M., Yamaguchi Y., Ryuno H., Hashimoto T. (2019). Experiences of violence and preventive measures among nurses in psychiatric and non–psychiatric home visit nursing services in Japan. J. Psychosoc. Nurs. Ment. Health Serv..

[bib0014] Gabrovec B. (2017). Prevalence of violence toward community nurses: a questionnaire survey. Workplace Health Saf..

[bib0015] Harris P.A., Taylor R., Minor B.L., Elliott V., Fernandez M., O'Neal L., McLeod L., Delacqua G., Delacqua F., Kirby J., Duda S.N. (2019). The REDCap consortium: building an international community of software platform partners. J. Biomed. Inform..

[bib0016] Harris P.A., Taylor R., Thielke R., Payne J., Gonzalez N., Conde J.G. (2009). Research electronic data capture (REDCap)—A metadata-driven methodology and workflow process for providing translational research informatics support. J. Biomed. Inform..

[bib0017] Hibino Y., Hitomi Y., Kambayashi Y., Nakamura H. (2009). Exploring factors associated with the incidence of sexual harassment of hospital nurses by patients. J. Nursing Scholarship.

[bib0018] Kim M., Kim T., Tilley D.S., Kapusta A., Allen D., Cho H.S.M. (2018). Nursing students’ experience of sexual harassment during clinical practicum: a phenomenological approach. Korean J. Women Health Nursing.

[bib0020] Koller J., Spörhase U. (2018). Die Herausforderung grenzüberschreitender Sexualität in der stationären Altenpflege. HeilberufeSci..

[bib0021] Lenhard W., Lenhard A. (2016). https://www.psychometrica.de/effektstaerke.html.

[bib0022] Leonhart R. (2010).

[bib0023] Lu L., Dong M., Lok G.K.I., Feng Y., Wang G., Ng C.H., Ungvari G.S., Xiang Y.-.T. (2020). Worldwide prevalence of sexual harassment towards nurses: a comprehensive meta-analysis of observational studies. J. Adv. Nurs..

[bib0024] Maghraby R.A., Elgibaly O., El-Gazzar A.F. (2020). Workplace sexual harassment among nurses of a university hospital in Egypt. Sexual & Reproductive Healthcare.

[bib0025] Magnavita N., Heponiemi T. (2011). Workplace violence against nursing students and nurses: an Italian experience. J. Nursing Scholarship.

[bib0026] Margavi M.K., Bagheri-Nesami M., Mousavinasab N., Lolaty H.A. (2020). Frequency of violence against nurses and its related factors during cardiopulmonary resuscitation in emergency wards. J. Nursing & Midwifery Sci..

[bib0027] McClendon S., Farbman R. (2018). https://www.nursingworld.org/news/news-releases/2018/ana-addresses-sexual-harassment-as-part-of-endnurseabuse-initiative/.

[bib0028] Nelson R. (2018). Sexual harassment in nursing: a long-standing, but rarely studied problem. Am. J. Nurs..

[bib0029] Nielsen M.B.D., Kjær S., Aldrich P.T., Madsen Ida.E.H., Friborg M.K., Rugulies R., Folker A.P. (2017). Sexual harassment in care work – Dilemmas and consequences: a qualitative investigation. Int. J. Nurs. Stud..

[bib0019] Kind + Spital. (2016). Die EACH Charta mit Erläuterungen. Retrieved November 21, 2021, from https://www.kindundspital.ch/download/pictures/b5/7y45twtm2ywb221wohdb04jovo7puw/eachcharta_de.pdf.

[bib0030] Pädiatrische Pflege Schweiz. (2020). Definition Pädiatrische Pflege. Retrieved November 25, 2021, from https://swisspediatricnursing.ch/definition-p%C3%A4diatrische-pflege-definition-pediatric-nursing-d%C3%A9finition-des-soins-infirmiers-p%C3%A9diatriques-definizione-di-cure-pediatriche/index.

[bib0003] Bundesamt für Statistik. (2018). Hochschulstatistik. Retrieved November 24, 2021, from https://www.bfs.admin.ch/bfsstatic/dam/assets/4582969/master.

[bib0031] Park M., Cho S.-.H., Hong H.-.J. (2015). Prevalence and perpetrators of workplace violence by nursing unit and the relationship between violence and the perceived work environment. J. Nursing Scholarship.

[bib0032] Schlup N., Gehri B., Simon M. (2021). Prevalence and severity of verbal, physical, and sexual inpatient violence against nurses in Swiss psychiatric hospitals and associated nurse-related characteristics: cross-sectional multicentre study. Int. J. Ment. Health Nurs..

[bib0033] Spector P.E., Zhou Z.E., Che X.X. (2014). Nurse exposure to physical and nonphysical violence, bullying, and sexual harassment: a quantitative review. Int. J. Nurs. Stud..

[bib0034] Staatssekretariat für Bildung, Forschung und Innovation, & Eidgenössisches Departement für Wirtschaft, Bildung und Forschung (2016). https://www.sbfi.admin.ch/dam/sbfi/de/dokumente/schlussbericht_masterplanbildungpflegeberufe.pdf.download.pdf/schlussbericht_masterplanbildungpflegeberufe.pdf.

[bib0035] Temkin S.M., Chapman-Davis E., Nair N., Cohn D.E., Hines J.F., Kohn E.C., Blank S.V. (2022). Creating work environments where people of all genders in gynecologic oncology can thrive: an SGO evidence-based review. Gynecol. Oncol..

[bib0036] Tsukamoto S.A.S., Galdino M.J.Q., Robazzi M.L.do C.C., Ribeiro R.P., Soares M.H., Haddad M.do C.F.L., Martins J.T. (2019). Occupational violence in the nursing team: prevalence and associated factors. Acta Paulista de Enfermagem.

[bib0037] Vincent-Höper S., Adler M., Stein M., Vaupel C., Nienhaus A. (2020). Sexually harassing behaviors from patients or clients and care workers’ mental health: development and validation of a measure. Int. J. Environ. Res. Public Health.

[bib0038] Zeng L.-.N., Lok K.-.I., An F.-.R., Zhang L., Wang D., Ungvari G.S., Bressington D.T., Cheung T., Chen L., Xiang Y.-.T. (2020). Prevalence of sexual harassment toward psychiatric nurses and its association with quality of life in China. Arch. Psychiatr. Nurs..

[bib0039] Zentrale Ausgleichsstelle (2019). https://www.zas.admin.ch/zas/de/home/particuliers/rentes-de-vieillesse.html.

